# Role of Ureteroscopy in Treatment of Upper Tract Urothelial Carcinoma

**DOI:** 10.1007/s11934-021-01065-7

**Published:** 2021-10-07

**Authors:** Jeremy Ng Chieng Hin, Dinul Hettiarachchilage, Paul Gravestock, Bhavan Rai, Bhaskar K. Somani, Rajan Veeratterapillay

**Affiliations:** 1 University of Newcastle, Newcastle, UK; 2 Department of Urology, Freeman Hospital, Newcastle, UK; 3 Department of Urology, University Hospital Southamptom, Southamptom, UK

**Keywords:** Ureteroscopy, Upper tract urothelial carcinoma, Adjuvant intracavitary therapy, Transitional cell carcinoma, Laser, Mitomcycin

## Abstract

**Purpose of Review:**

Upper tract urothelial carcinoma (UTUC) is uncommon accounting for less than 10% of all urothelial tumours. Ureteroscopic management (URS) is the first line treatment for low-risk disease and has been increasingly utilised due to technological advances and increasing surgical experience. This review looks at patient outcomes relating to URS, emerging technologies and the role of adjuvant intracavitary therapy in the management of UTUC.

**Recent Findings:**

URS has firmly established itself in the management algorithm for UTUC, and a good body of evidence supports its use for low-risk disease, wherein oncological outcomes are comparable to traditional nephroureterectomy (RNU). Larger tumours can now be managed using URS with a lower morbidity than radical surgery, though with higher associated local recurrence rate and risk of progression to RNU, and as a result, patient selection and close surveillance remains key. There is limited evidence for adjuvant intracavitary therapy (Mitomycin C or BCG) in UTUC although the development of novel polymers and biodegradable stents may improve drug delivery to the upper urinary tract.

**Summary:**

URS has a clearly defined role in low-risk UTUC, and its use in larger tumours appears to be appropriate in a selected cohort of patients. The efficacy of adjuvant intracavitary therapy is as of yet undetermined, though developments in delivery techniques are promising. Likewise further developments of laser technology are anticipated to further expand the role of URS.

**Supplementary Information:**

The online version contains supplementary material available at 10.1007/s11934-021-01065-7.

## Introduction

Upper tract urothelial carcinoma (UTUC) is uncommon accounting for less than 10% of all urothelial tumours. At diagnosis, 60% of UTUC are found to be invasive, and radical nephroureterectomy (RNU) has traditionally been the gold standard treatment [1]. Over the past three decades, there has been accumulating experience for the use of endoscopic management in selected cases of UTUC. Endoscopic management in UTUC can be via either a percutaneous or ureteroscopic approach. Ureteroscopic management (URS) has been increasingly utilised over the percutaneous route, and this has been due to a number of factors which include improved ureteroscope design, the advent of flexible instruments and advances in laser technology. These technological factors are coupled with increased experience and dissemination of the surgical techniques driven by concentration of cases in large-volume surgical units [2]. Whilst early experience of ureteroscopic treatment of UTUC was limited to specific patient groups such as bilateral UTUC, solitary renal unit or established chronic kidney disease, the indications for endoscopic management have been expanded over time.

The most recent EAU guidelines for UTUC published in 2020 recommends kidney sparing treatment for all low-risk tumours, with ureteroscopic management as first line for lesions that would be amenable to it [3]. Low-risk UTUC is defined as unifocal disease, tumour size of less than 2 cm, low-grade cytology, low grade on ureteroscopic biopsy and no invasive aspect seen on cross sectional imaging [3]. The rationale for ureteroscopic management in low-risk patients is that overall survival outcomes may be superior to RNU owing to the preservation of renal function and reduced surgical morbidity. However, close surveillance is required for patients managed endoscopically as recurrences and progression to RNU are a well-documented occurrence.

We discuss the expanding role of URS for UTUC with a focus on oncological outcomes, intracavitary therapy, modality of tumour ablation and emerging technologies.

## Literature Search

A literature search was undertaken of the Medline and Embase online databases for English language publications within the period 01 Jan. 2000-30 Aug. 2020. Search terms used were ‘upper tract urothelial carcinoma’, upper tract transitional cell carcinoma’, ‘UTUC’ combined with ‘endoscopic management’ or ‘ureteroscopy’ or ‘URS’, or ‘RIRS’ or ‘retrograde intrarenal surgery’, or ‘laser ablation’ or ‘nephroureterectomy’ or ‘mitomycin’, or ‘MMC’ or ‘BCG’ or ‘intravesical recurrence’. Papers were identified and were screened independently by two authors (NJ + HD). Studies describing survival outcomes with < 40 patients were excluded. For studies describing intracavitary therapy, a minimum of 10 patients was required for inclusion. Forty papers were included for final analysis, and relevant studies were synthesised for a narrative review.

### Inclusion Criteria

1. All studies in English language reporting on patients undergoing endoscopic treatment for UTUC
2. Intracavitary treatment using MMC or BCG

### Exclusion Criteria

1. Non-English language articles
2. Narrative review articles, case reports, laboratory or animal studies
3. Grey literature and studies where outcome of interest was not presented

**Figure 1:**
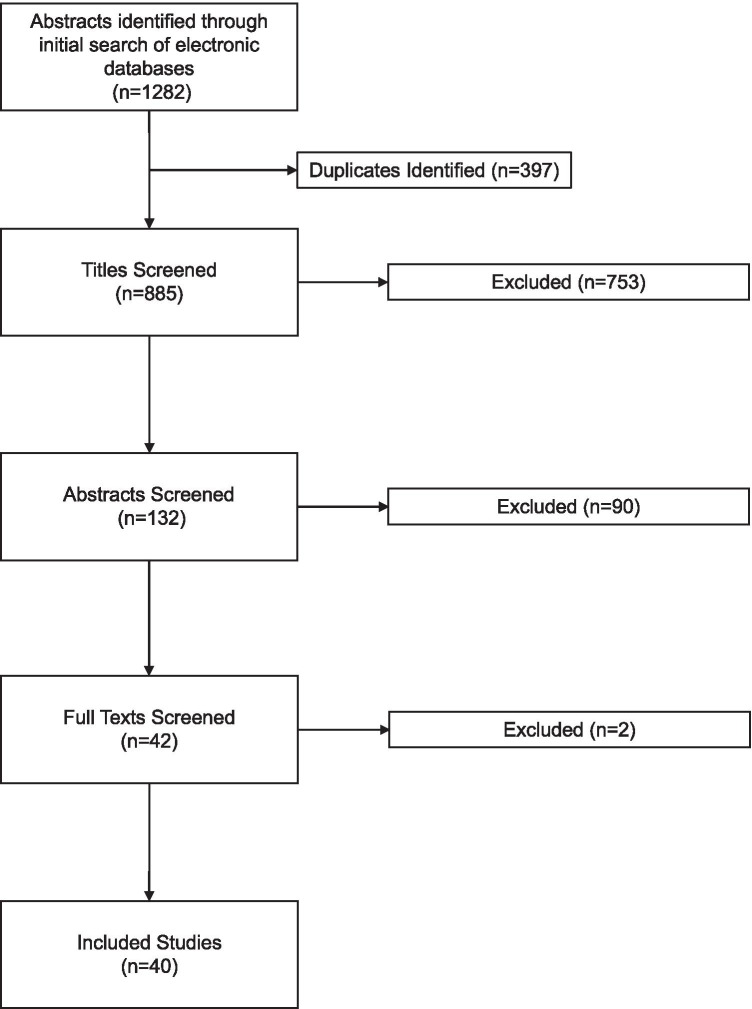
PRISMA diagram outlining article selection process

Search results were summarised within a Preferred Reporting Items in Systematic Reviews and Meta-Analyses (PRISMA) flow chart (Fig. 1) [4].

## Comparative Outcomes of URS and RNU

There has been a trend towards increased utilisation of URS for UTUC over the past two decades. This was highlighted in an analysis of the National Cancer Database (NCDB) over a 10-year period (2004–2013) which revealed that the rate of endoscopic ablation increased from 9.8 to 11.5% whilst the overall rate of RNU decreased from 59.6 to 56.7% [5]. In addition, an analysis over a similar time period found that patients who were treated at an academic facility, who were older and had fewer comorbidities were more likely to receive endoscopic treatment instead of RNU [6].

There are only a small number of studies comparing outcomes of URS to RNU which include no randomised controlled trials, and therefore, the majority of the included studies were retrospective case series or comparative studies (Table 1) [7–25, 26•]. Results from comparative studies should be interpreted with caution as patients undergoing URS often have imperative indications for this approach (such as solitary kidney or bilateral tumours), are often carefully selected and likely to be of lower stage. In addition, the definitive pathological stage for patients managed exclusively by URS is not always available, thereby often preventing direct comparison to patients undergoing RNU. Additionally, the distribution of invasive UTUC stage (≥ pT2) in the RNU study arms was significantly higher in the studies that reported this data (up to 67% for RNU vs 24% for URS) further skewing the analysis.

**Table 1:** Summary of studies reporting URS outcomes in UTUC

		**Number of patients**		**Median age (yrs)**		**Follow Up (mo)**	**Ablation energy**	**5-year CSS**^**d**^** (%)**		**Progression to NU**^**e**^** (%)**	**Recurrence (%)**		**Complications**
											**UT**^f^	**Bladder**	
**Author**	**Year**	*URS*	*RNU*	*URS*	*RNU*	*Median*	*(in URS group)*	*URS*	*RNU*	*URS group*			
**Comparative studies (URS vs RNU)**													
Roupret et al.[7]	2006	27	54	68		57.5	Ho:YAG^g^	80.7%	84%	25.9%	44%	22.2%	11%
Lucas et al. [8]	2008	39	77	68	65	46	Ho:YAG	81.6%	83%	28.2%	44%	10%	–
Gurbuz et al. [9]^a^	2011	175	1093	71.2	69.2	52.8	–	77%	73%	100%^a^	–	–	–
Grasso et al. [10]	2012	66	80	73	72.5	51.5	Ho:YAG/Nd:YAG^h^	LG 87%	64% (LG 93%)	16.6%	81%	60.6%	–
Cutress et al. [11]	2012	73	–	69.3		54	Nd:YAG	88.9%	–	19.2%	68.5%	42.5%	19%
Murray et al. [12]^a^	2015	167	838	>65 years		96	–	HR 1.69^‡^ *(worse in URS)*		100%^a^	–	–	–
Vemana et al. [13]	2016	151	302	75		43	–	88%	92%	–	–	–	–
Bozzini et al. [14]	2020	47	31			11.7	Thu:YAG^i^	–	–	–	19.2%	–	–
**Non-comparative (URS only)**													
Deligne et al. [15]	2002	61		66.2		39.9	Ho:YAG/Nd:YAG	77%		19%	24.6%	14.8%	2 ureteric strictures
Krambeck et al. [16]	2007	78		73		52	KTP^j^/Ho:YAG/Nd:YAG	71.2%		35.9%	–	49.3%	17.8%
Sowter et al. [17]	2007	40		65		41.6	Ho:YAG/Nd:YAG	–		29.3%	74.3%	–	–
Thompson et al. [18]	2008	83		71		55	Nd:YAG	85.4%		32.5%	55.4%	44.6%	–
Pak et al. [19]	2009	57		65.6		53	–	94.7%		19%	89.5%	–	–
Nita et al. [20]	2012	65		67		60	Nd:YAG	–		27.7%	47.7%	30.7%	–
Vanacore et al. [21]^b^	2016	186		72.6		24	Ho:YAG/Thu:YAG	–		13.50%	49%	–	CD^k^ ≥ III 0%
Scotland et al. [22]^c^	2018	80		75.2		43.6	Ho:YAG/Nd:YAG	84%		20%	90.5%	30.2%	–
Defidio et al. [23]	2018	60		–		24.4	–	77.5%		–	71.4%	–	–
Musi et al. [24]	2018	42		68		26.3	Thu:YAG	–		9.5%	19%	–	CD I 38%, II 46%, III 3%
Defidio et al. [25]	2019	101		71.1		28.7	Ho:YAG/Thu:YAG	–		8.9%	30.7%	–	CD I 10%
Scotland et al. [26•]	2020	168		70		66	Ho:YAG/Nd:YAG	92.6%		29.8%	71.4%	–	7.1%

^a^Compared immediate to delayed RNU

^b^Confocal laser microscopy performed in 23 patients

^c^Large tumours only (> 2 cm)

^d^Cancer-specific survival

^e^Nephroureterectomy

^f^Upper tract

^g^Holmium

^h^Neodymium-doped

^i^Thulium

^j^Potassium titanyl phosphate

^k^Clavien Dindo

Across the included studies, 5-year cancer-specific survival (CSS) for URS was 71.2–94.7% compared to 64–92% in the RNU group. In patients managed by URS, there was a progression rate of 8.9–53% to RNU. There are conflicting reports on the survival outcomes of patients undergoing delayed RNU following initial ureteroscopic management. Two studies utilising Surveillance, Epidemiology and End Results (SEER) data compared patients who underwent a primary RNU to those who had initial endoscopic treatment followed by RNU. The first compared 838 patients who underwent RNU with 167 who had undergone RNU following initial endoscopic treatment and found a significantly worse 5-year CSS for the latter group (HR 1.69, 95%CI 1.07–2.69) months but noted no significant difference in overall survival (OS) [6]. The second, a propensity score analysis of 453 low-grade UTUC patients, reported that whilst the delayed RNU group had similar results within the first 24 months, after this period, survival curves diverged significant, with inferior OS and CSS in those who had received initial endoscopic management. Furthermore, the CSS in those who had received delayed RNU remained similar to those who had undergone endoscopic management alone [13].

On the other hand, Gurbuz et al. analysed an international multicentre database and reported no difference in 5-year CSS in patients having a deferred RNU following endoscopic intervention (*n* = 175) compared to primary RNU (*n* = 1093), 77% vs 73% respectively (*p* = 0.365)[9]. Furthermore, history of ablation therapy was not associated with cancer-specific mortality (HR 0.79 [0.55–1.22] *p* = 0.185) [9].

Overall, whilst acknowledging the limitations of reported studies, URS outcomes may be similar in the short-term to RNU in carefully selected low-grade tumours. The rate of progression to RNU remains high though, and close surveillance is therefore vital.

## Survival Outcomes Post-ureteroscopic Ablation

URS allows diagnostic evaluation and primary tumour ablation with the advantages of renal preservation and lower surgical morbidity compared with RNU. The majority of these studies are retrospective single-centre series with some reporting analyses of national datasets due to the rarity of the condition. Analysis of included studies shows patient numbers ranging between 40 and 186 with a median age of 65–75.2 years. There was a wide variation in follow-up, between 11.7 and 96 months. Five-year overall survival ranged between 57 and 75%, and 5-year cancer-specific survival was 64–94.7% across the included studies (Table 1). Scotland et al. reported on one of the largest retrospective series of URS in 168 patients, reporting a mean tumour size of 16.8 mm and found a 5-year CSS of 92.6% and recurrence-free survival of 30% [26•].

It is worth noting that the reported CSS across studies may be overestimates as histological confirmation was not universally performed and that up to 16% of visually suspected UTUC could harbour benign pathology [27]. Metastatic progression is uncommonly reported, but previous reports estimated a pooled 9% rate following URS with a 5-year metastatic-free survival of 94% for low-risk disease [27].

## Upper Tract Recurrence Post-ureteroscopic Management

Recurrence following initial URS tumour ablation was 19–90.5%, and progression to RNU was noted in 8.9–53% (Table 1). A number of studies have examined risk factors predicting recurrence post URS or progression to RNU. Mohapatra et al. reported on a series of 170 patients across two institutions where 89 patients progressed to RNU. They found that ureteroscopic visualisation, biopsy grade and positive urine cytology were higher in those progressing to RNU. In addition, they calculated that the probability of not undergoing RNU was 50% at 2 years and 20% at 5 years post URS [28]. Tumour grade was also noted to achieve predictor status for recurrence-free survival in another cohort of 41 patients managed endoscopically [29].

Tumour size is an important criterion affecting the success of URS. Defidio et al. reported a worse 5-year disease-specific survival (DSS) for UTUC >1 cm managed endoscopically (93% vs 67%) in a cohort of 60 patients [23]. Another cohort study of 92 patients managed with holmium/yttrium-aluminium-garnet (Ho:YAG) laser with a median follow-up of 52 months reported no statistical difference in progression-free survival with tumour size of < 1 cm compared to > 1 cm (68% vs. 72%). The only independent predictor of disease progression in this study was tumour grade at initial biopsy [30•]. Scotland et al. reported a retrospective cohort study of 80 patients (median FU 43.6 months) with low-grade UTUC > 2 cm managed by URS with 5-year CSS was 84%. However, 90.5% tumours recurred and 20% required RNU [22]. A more recent report involving 343 URS in 87 patients with UTUC reported a local recurrence rate of 46% at a mean follow-up time of 4.9 months after initial URS in tumours > 2 cm compared to 71% after a mean follow-up time of 9.9 months in tumours < 2 cm. This suggests that larger tumours tend to recur faster locally but only one patient in the larger tumour group required RNU after 12 months of URS management [31].

Tumour location and prior history of bladder cancer have also been associated with higher local recurrence rates in other reports although this has not been consistently documented [20, 27]. Overall, the data would suggest that tumour size and grade remain important predictors of initial success of URS. Whilst larger tumours (> 2 cm) can be treated with URS, early recurrence rates can be high, and close endoscopic follow-up remains vital.

## Intravesical Recurrence Rates Post-URS

Intravesical recurrence has been reported between 10 and 60.6% of the included studies at a median follow-up time of 46–60 months. The previous pooled incidence of intravesical recurrence was reported at 34% [27]. Although intravesical recurrence is dependent on several confounding factors, including prior history of bladder cancer, tumour grade and stage, it has been suggested that the performance of URS in UTUC could also be an independent predictor for this. In a meta-analysis of 2382 patients, intravesical recurrence rates were 39.2–60.7% in patients with prior URS compared to 16.7–46% in patients who did not undergo URS but proceeded directly to RNU [32]. In the pooled analysis, a statistically significant association was found between performance of URS prior to RNU and intravesical recurrence rates.

## Modality of Laser Ablation and Emerging Technologies

The holmium/yttrium-aluminium-garnet (Ho:YAG) and neodynium/yttrium-aluminium-garnet (Nd:YAG) lasers have been extensively described in the management of UTUC. The Ho:YAG laser energy is readily absorbed by water and must be used in contact with the tissue to achieve tumour ablation. The Nd:YAG laser has a greater depth of penetration (4–6 mm) and provides a deeper coagulation and ablative effect on the tumour. More recently, thulium/YAG (Thu:YAG) ablation of UTUC has been described in a number of series. Thu:YAG provides a continuous wave and a lower tissue penetration resulting in good vaporisation and coagulation properties for treating soft tissue disease, though it does appear to cause more tissue necrosis [33]. Four studies included in our review used Thu:YAG, two in combination with Ho:YAG and two in isolation [14, 21, 24, 25].

The included studies using Ho:YAG or Nd:YAG showed progression to NU in 16.6–35.9% vs 8.9–13.5% in studies using Thu:YAG and upper tract recurrence 24.6–90.5% vs. 19.2–49% for Thu:YAG [14, 21, 24, 25]. Defidio et al (2011) reported the comparative outcomes of Thu:YAG and Ho:YAG in a multicentre European study. They studied 59 patients and reported non-inferior recurrence-free survival for Thu:YAG. They however reported reduced bleeding and mucosal perforation rates and improved ablation efficiency in tumours < 1.5 cm. There was no difference in ablation efficiency with Thu:YAG for tumours > 1.5 cm [34]. The largest single-centre experience of Thu:YAG described outcomes of 42 patients with a median age of 68 years and median follow-up of 26.3 years. Upper tract recurrence was 19%, and 9.5% progressed to NU. The major complication rate (Clavien > 3) was 2.4% [24].

Confocal laser endomicroscopy (CLE) technology is being assessed for its use in UTUC. The technology would allow improved detection of tumours at the time of URS, and this may lead to improved tumour ablation. Vanacore et al. studied 186 pts undergoing URS with CLE (Cellvizio) technology and ablation using combination of Ho:YAG and Thu:YAG. They reported a 49% upper tract recurrence rate and 13.5% progression rate to RNU, though CLE was only used 23 of the 186 patients [21]. The novel thulium fibre laser (TFL) is emerging as a promising tool in endourological management and has advantages of better energy absorption, limited penetration and limited carbonisation thereby producing a limited ablation zone [35, 36]. Martov et al. (2018) reported their early experience of TFL in a small cohort of 11 patients with UTUC with size of 1–4.5cm. They reported no major complications and no recurrence at 6 months [37•]. New technologies hold the promise of improving oncologic outcomes of patients selected for endoscopic management of UTUC although larger datasets are required to confirm the findings.

## Complications and Surveillance Post-endoscopic Treatment

Complications were not uniformly reported between studies and varied significantly in the studies that did provide data, ranging from 7.1 to 46% (Table 1) with the commonest serious complication reported being ureteric stricture. The incidence of ureteric stricture post URS has been reported to be between 0 and 27%.[38]. Serious and fatal complications following URS are rare, and ureteroscopic management of UTUC is safe in appropriately selected patients.

Due to the risk of upper tract recurrence, intravesical recurrence and progression rate to RNU, close surveillance is required following initial URS. Surveillance should involve both the upper tracts and the bladder. The current EAU guidelines recommend a risk-stratified approach to follow-up [3]. In low-risk tumours, a check URS is recommended at 3 months after initial endoscopic management as well as a cystoscopy and computed tomography (CT) urogram at 3 months, 6 months and then annually for 5 years. For high-risk tumours, URS and in situ cytology at 3 and 6 months are recommended following initial endoscopic therapy and in addition cystoscopy, urine cytology and CT (urogram and chest) at 3 and 6 months then annually thereafter [3].

## Role of Intracavitary Therapy

Whilst the role of adjuvant intracavitary therapy is well established in urothelial carcinoma of the bladder, the data for its use in UTUC is limited. A number of agents have been utilised in this context, extrapolating from the experience in urothelial bladder cancer including Bacille-Calmette-Guerin (BCG) and mitomycin C (MMC). The data for contemporary studies reporting a minimum of 10 patients is summarised in Table 2 [11, 39–47].

**Table 2:** Summary of studies looking at the outcomes of adjuvant treatment for UTUC

**Author**	**Year**	**Patients (*n*)**	**Median FU**^**a**^	**Pathology**	**Drug**	**CR**^**b**^** (BCG)**	**Recurrence (%)**	**5-yr RFS**^**c**^	**Progression (CIS**^**d**^**)**
Nonomura et al. [39]	2000	11	20*	CIS	BCG^e^	82%	27%	–	27%
Goel et al. [40]	2003	10	64	Ta/T1	MMC^f^		53%	–	–
Palou et al. [41]	2004	19	51*	Ta/T1	BCG/MMC		58%	–	–
Kojima et al. [42]	2006	11	58	CIS	BCG	82%	27%	78%	18%
Rastinehad et al. [43]	2009	50	40	Ta/T1	BCG		36%	–	–
Giannarini et al. [44]	2011	18 (22 RU^g^)	42	Ta/T1	BCG	–	59%	–	–
Giannarini et al. [44]	2011	37 (42 RU)	42	CIS	BCG	–	40%	57%	5%
Cutress et al. [11]	2012	18	54	Ta/T1	MMC	–	–	54%	–
Shapiro et al. [45]	2012	11	14	CIS	BCG	73%	11%	–	0%
Horiguchi et al. [46]	2018	38	49	CIS	BCG	79%	–	–	43%
Tomisaki et al. [47]	2018	41 (52 RU)	26	CIS	BCG	90%	23%	60%	13%

*Mean

^a^Follow up

^b^Complete response

^c^Recurrence-free survival

^d^Carcinoma in situ

^e^Bacillus Calmette–Guérin

^f^Mitomycin C

^g^Renal units

All studies had a small number of patients and a variable follow-up (median 13.5–64 months). Many of the studies consisted of larger cohorts with only a small subgroup receiving adjuvant treatment and outcomes specific to adjuvant therapy often not well reported. The largest study by Rastinehad et al. (2009) reported outcomes of 50 patients with a median FU of 40.8 months who had had adjuvant BCG for Ta/T1 tumour. They reported upper tract recurrence rates of 36% and CSS of 98%, though the difference compared with those who did not receive adjuvant BCG was not statistically significant with a recurrence rate of 30.7% (*n* = 39)[43]. The study by Goel et al. (2003) had the longest FU with a median of 64 months and included patient with Ta/T1 tumours treated with adjuvant MMC. The authors reported a 53% recurrence rate and 88% CSS (39).

Foerster et al. (2019) reported a meta-analysis of topical treatments for UTUC which included 438 patients [48]. Topical treatment was delivered via a retrograde or antegrade approach with studies utilising BCG, MMC or a combination of both. For patients with Ta/T1 UTUC, overall pooled estimates showed that the rate of upper tract recurrence was 40%, of CSS was 94% and overall survival of 71%. Sub-analyses stratified by drug use and instillation approach did not show significant differences. For primary CIS treated with topical BCG following endoscopic management, the pooled estimate for upper tract recurrence was 34% and progression of 16%. Comparison between instillation approaches again did not show any statistically significant differences [48]. These data suggest that there may be limited benefit to intracavitary therapy as the reported recurrence and survival outcomes are similar to those groups of patients managed by observation alone [48].

Gallioli et al. reported a preliminary study on the use of adjuvant single-dose mitomycin (ASDM) immediately after therapeutic URS for UTUC in a cohort of 52 patients. They found a reduction in urothelial recurrence rate (23.5% vs. 55.5% if ASDM was not used) although recurrence-free survival rates were not statistically significant at a median follow-up time of 18 months [49]. The effect of second line topical therapy of UTUC was investigated in a cohort study of 51 patients. Response rates were 71% following first-line topical treatment and 62% to second-line treatment for patients having adjuvant therapy for Ta/T1 tumours suggesting that this could be a useful strategy [50].

It is to be noted that the efficacy of intracavitary therapy may be hampered by the dwell time as it is difficult to keep the required agent in the upper tract due to natural drainage. Traditional treatment regimes have been based on continuous drug delivery controlled through a pump either retrograde via a ureteric catheter or antegrade via a nephrostomy. However, one possible solution is the development of a novel polymer with reverse thermal reaction combined with mitomycin (Mitogel). Uniquely, it exists as a liquid at cold temperature but becomes a viscous gel at body temperature and can remain in the upper tracts for 4–6 h thereby theoretically improving drug delivery. Initial results of the trial, published in The Lancet, evaluating the use of this compound for chemoablation of low risk UTUC showed encouraging results, reporting a 59% rate of complete response and partial response in a further 11% [51••]. Another promising development is biodegradable drug–eluting stents which can be impregnated with chemotherapeutic agents for improved drug delivery into the upper urinary tract. In vitro studies have shown a reduction of up to 75% in cancer cell lines exposed to biodegradable stents, and further studies are required to confirm this [52].

## Areas of Future Research

Although previous review suggested a paucity of evidence in management of UTUC, it seems that recent evidence and guidelines show a growing role of ureteroscopy, especially for low-risk disease [53]. Laser technique, technology and ancillary equipment will also help in facilitating this [54]. This has to be balanced with the cost of procedure and the risk of recurrent or progressive disease [55].

## Conclusions

Ureteroscopic management has firmly established itself in the management algorithm for UTUC. A good body of evidence supports its use for low-risk UTUC where oncological outcomes are comparable to traditional nephroureterectomy.

With increased experience and with the development of ureteroscopic and ablation energy technology, it is now possible to treat tumours of larger size with lower morbidity compared to traditional nephroureterectomy. However, this comes at the cost of higher local recurrence rates and progression to radical surgery, and therefore patient selection and close surveillance remains key. There is limited evidence for adjuvant intracavitary therapy in UTUC although the development of novel polymers and biodegradable stents may improve drug delivery to the upper urinary tract. Early reports of the novel thulium fibre laser are encouraging, and this could radically change the landscape of ureteroscopic management of UTUC.

## Supplementary Information

Below is the link to the electronic supplementary material.

Supplementary file1 (DOCX 13 KB)

Supplementary file2 (PDF 1705 KB)

Supplementary file3 (DOCX 24 KB)

Supplementary file4 (DOCX 24 KB)

Supplementary file5 (DOCX 24 KB)

Supplementary file6 (DOCX 24 KB)

Supplementary file7 (DOCX 24 KB)

Supplementary file8 (DOCX 28 KB)

Supplementary file9 (PDF 93 KB)

## Data Availability

Not applicable.

## References

[1] Szarvas T, Modos O, Horvath A (2016). Why are upper tract urothelial carcinoma two different diseases?. Transl Androl Urol..

[2] Leow JJ, Liu Z, Tan TW (2020). Optimal management of upper tract urothelial carcinoma: current perspectives. Onco Targets Ther..

[3] Roupret M, Babjuk M, Burger M (2021). European Association of Urology Guidelines on Upper Urinary Tract Urothelial Carcinoma: 2020 update. Eur Urol..

[4] Moher D, Liberati A, Tetzlaff J, et al. The PRISMA Group. Preferred Reporting Items for Systematic Reviews and Meta-Analyses: The PRISMA Statement. Int J Surg 2010; doi:10.1016/j.ijsu.2010.02.007 PMC3090117 21603045

[5] Browne BM, Stensland KD, Moynihan MJ (2018). An analysis of staging and treatment trends for upper tract urothelial carcinoma in the National Cancer Database. Clin Genitourin Cancer..

[6] Upfill-Brown A, Lenis AT, Faiena I (2019). Treatment utilization and overall survival in patients receiving radical nephroureterectomy versus endoscopic management for upper tract urothelial carcinoma: evaluation of updated treatment guidelines. World J Urol..

[7] Roupret M, Hupertan V, Traxer O (2006). Comparison of open nephroureterectomy and ureteroscopic and percutaneous management of upper urinary tract transitional cell carcinoma. Urology..

[8] Lucas SM, Svatek RS, Olgin G (2008). Conservative management in selected patients with upper tract urothelial carcinoma compares favourably with early radical surgery. BJU Int..

[9] Gurbuz C, Youssef RF, Shariat SF (2011). The impact of previous ureteroscopic tumor ablation on oncologic outcomes after radical nephrouretectomy for upper urinary tract urothelial carcinoma. J Endourol..

[10] Grasso M, Fishman AI, Cohen J (2012). Ureteroscopic and extirpative treatment of upper urinary tract urothelial carcinoma: a 15-year comprehensive review of 160 consecutive patients. BJU Int..

[11] Cutress ML, Stewart GD, Wells-Cole S (2012). Long-term endoscopic management of upper tract urothelial carcinoma: 20-year single-centre experience. BJU Int..

[12] Murray K, Winer A, Bagrodia A, et al. Endoscopic management for upper tract urothelial cancer (UTUC) compared to immediate nephroureterectomy: survival outcomes in seer database and cancer center cohort. Journal of Urology. 2015;193(4).

[13] Vemana G, Kim EH, Bhayani SB (2016). Survival Comparison between endoscopic and surgical management for patients with upper tract urothelial cancer: a matched propensity score analysis using surveillance, epidemiology and end results-Medicare data. Urology..

[14] Bozzini G, Gastaldi C, Besana U, et al. Thulium-laser retrograde intra renal ablation (T-RIRA) of upper urinary tract transitional cell carcinoma: an ESUT study. Minerva Urol Nefrol. 2020. 10.23736/S2724-6051.20.03689-9 32026668

[15] Deligne E, Colombel M, Badet L (2002). Conservative management of upper urinary tract tumors. Eur Urol..

[16] Krambeck AE, Thompson RH, Lohse CM (2007). Endoscopic management of upper tract urothelial carcinoma in patients with a history of bladder urothelial carcinoma. The Journal of urology.

[17] Sowter SJ, Ilie CP, Efthimiou I (2007). Endourologic management of patients with upper-tract transitional-cell carcinoma: long-term follow-up in a single center. J Endourol..

[18] Thompson RH, Krambeck AE, Lohse CM (2008). Endoscopic management of upper tract transitional cell carcinoma in patients with normal contralateral kidneys. Urology..

[19] Pak RW, Moskowitz EJ, Bagley DH (2009). What is the cost of maintaining a kidney in upper-tract transitional-cell carcinoma? An objective analysis of cost and survival. J Endourol..

[20] Nita G, Georgescu D, Multescu R (2012). Prognostic factors in laser treatment of upper urinary tract urothelial tumours. J Med Life..

[21] Vanacore D, Sanguedolce F, Montano B, et al. Evolving techniques of endoscopic UTUC management: optimising outcomes with the appropriate use of latest technologies. Journal of Urology. 2020;203.

[22] Scotland KB, Kleinmann N, Cason D (2018). Ureteroscopic management of large >/=2 cm upper tract urothelial carcinoma: a comprehensive 23-year experience. Urology..

[23] Defidio LD, De Dominicis M, Calarco A, et al. Is it possible and safe to treat endoscopically upper tract urothelial carcinoma larger than one centimeter? European Urology Supplements. 2018;17(4).

[24] Musi G, Mistretta FA, Marenghi C (2018). Thulium laser treatment of upper urinary tract carcinoma: a multi-institutional analysis of surgical and oncological outcomes. J Endourol..

[25] Defidio L, Antonucci M, De Dominicis M (2019). Thulium-Holmium:YAG duo laser in conservative upper tract urothelial cancer treatment: 13 years experience from a Tertiary National Referral Center. J Endourol..

[26] • Scotland KB, Hubbard L, Cason D, et al. Long term outcomes of ureteroscopic management of upper tract urothelial carcinoma. Urol Oncol. 2020;38(11):850 e17- e26. **Recent large study with long-term outcomes of ureterosopic management of upper tract tumours.** 10.1016/j.urolonc.2020.06.027 32773230

[27] Cutress ML, Stewart GD, Zakikhani P (2012). Ureteroscopic and percutaneous management of upper tract urothelial carcinoma (UTUC): systematic review. BJU Int..

[28] Mohapatra A, Strope SA, Vetter J, et al. Importance of long-term followup after endoscopic management for upper tract urothelial carcinoma and factors leading to surgical management. Can Urol Assoc J. 2017;11(9). 10.1007/s11255-020-02439-5 PMC7572076 32157621

[29] Villa L, Cloutier J, Letendre J (2016). Early repeated ureteroscopy within 6–8 weeks after a primary endoscopic treatment in patients with upper tract urothelial cell carcinoma: preliminary findings. World J Urol..

[30] Villa L, Haddad M, Capitanio U (2018). Which patients with upper tract urothelial carcinoma can be safely treated with flexible ureteroscopy with Holmium:YAG laser photoablation? Long-Term Results from a High Volume Institution. J Urol..

[31] Shvero A, Zilberman D, Laufer M, et al. Endoscopic treatment for upper urinary tract urothelial carcinoma-does size matter? J Urol. 2019;201(4). 10.1097/JU.0000000000001505 33216692

[32] Marchioni M, Primiceri G, Cindolo L (2017). Impact of diagnostic ureteroscopy on intravesical recurrence in patients undergoing radical nephroureterectomy for upper tract urothelial cancer: a systematic review and meta-analysis. BJU Int..

[33] Dolowy L, Krajewski W, Dembowski J (2015). The role of lasers in modern urology. Cent European J Urol..

[34] Defidio L, De Dominicis M, Di Gianfrancesco L (2011). First collaborative experience with thulium laser ablation of localized upper urinary tract urothelial tumors using retrograde intra-renal surgery. Arch Ital Urol Androl..

[35] Kronenberg P, Traxer O (2019). The laser of the future: reality and expectations about the new thulium fiber laser-a systematic review. Transl Androl Urol..

[36] Schembri M, Sahu J, Aboumarzouk O (2020). Thulium fiber laser: the new kid on the block. Turk J Urol.

[37] • Martov AG, Ergakov DV, Solomatnikov I, et al. Thulium superpulse fiber laser (TSPFL) in the endourological management of upper urinary tract urothelial carcinoma (UTUC). Journal of Endourology. 2018;32. **Recent paper using thulium fiber laser on endourological management of upper tract tumours.**

[38] Linehan J, Schoenberg M, Seltzer E (2021). Complications associated with ureteroscopic management of upper tract urothelial carcinoma. Urology..

[39] Nonomura N, Ono Y, Nozawa M, et al. Bacillus Calmette-Guerin perfusion therapy for the treatment of transitional cell carcinoma in situ of the upper urinary tract. Eur Urol. 2000;38(6):701-4;discussion 5. 10.1159/000020365 11111187

[40] Goel MC, Mahendra V, Roberts JG. Percutaneous management of renal pelvic urothelial tumors: long-term followup. J Urol. 2003;169(3):925-9; discussion 9-30. 10.1097/01.ju.0000050242.68745.4d 12576814

[41] Palou J, Piovesan LF, Huguet J (2004). Percutaneous nephroscopic management of upper urinary tract transitional cell carcinoma: recurrence and long-term followup. J Urol..

[42] Kojima Y, Tozawa K, Kawai N (2006). Long-term outcome of upper urinary tract carcinoma in situ: effectiveness of nephroureterectomy versus bacillus Calmette-Guerin therapy. Int J Urol..

[43] Rastinehad AR, Ost MC, Vanderbrink BA (2009). A 20-year experience with percutaneous resection of upper tract transitional carcinoma: is there an oncologic benefit with adjuvant bacillus Calmette Guerin therapy?. Urology..

[44] Giannarini G, Kessler TM, Birkhauser FD (2011). Antegrade perfusion with bacillus Calmette-Guerin in patients with non-muscle-invasive urothelial carcinoma of the upper urinary tract: who may benefit?. Eur Urol..

[45] Shapiro EY, Lipsky MJ, Cha DY (2012). Outcomes of intrarenal Bacillus Calmette-Guerin/interferon-alpha2B for biopsy-proven upper-tract carcinoma in situ. J Endourol..

[46] Horiguchi H, Yoneyama T, Hatakeyama S (2018). Impact of bacillus Calmette-Guerin therapy of upper urinary tract carcinoma in situ: comparison of oncological outcomes with radical nephroureterectomy. Med Oncol..

[47] Tomisaki I, Kubo T, Minato A (2018). Efficacy and tolerability of Bacillus Calmette-Guerin therapy as the first-line therapy for upper urinary tract carcinoma in situ. Cancer Invest..

[48] Foerster B, D'Andrea D, Abufaraj M (2019). Endocavitary treatment for upper tract urothelial carcinoma: a meta-analysis of the current literature. Urol Oncol..

[49] Gallioli A, Boissier R, Territo A, et al. Adjuvant single-dose upper urinary tract instillation of mitomycin C after therapeutic ureteroscopy for upper tract urothelial carcinoma: a single-centre 10.1089/end.2019.0750 32164441

[50] Balasubramanian A, Metcalfe M, Wagenheim G, et al. Results of second line topical therapy for upper tract urothelial carcinoma (UTUC). Eur Urol Suppl. 2017;16(3).

[51] Kleinmann N, Matin SF, Pierorazio PM (2020). Primary chemoablation of low-grade upper tract urothelial carcinoma using UGN-101, a mitomycin-containing reverse thermal gel (OLYMPUS): an open-label, single-arm, phase 3 trial. The Lancet. Oncology.

[52] Barros AA, Browne S, Oliveira C (2016). Drug-eluting biodegradable ureteral stent: new approach for urothelial tumors of upper urinary tract cancer. Int J Pharm..

[53] Rai BP, Shelley M, Coles B (2012). Surgical management for upper urinary tract transitional cell carcinoma (UUT-TCC): a systematic review. BJUI.

[54] Chapman RA, Somani BK, Robertson A (2014). Decreasing cost of flexible ureterorenoscopy: single use laser fiber cost analysis. Urology.

[55] Somani BK, Robertson A, Kata SG (2011). Decreasing the cost of flexible ureterorenoscopic procedures. Urology.

