# Supplementary material for: Role of Ureteroscopy in Treatment of Upper Tract Urothelial Carcinoma
Source: Curr Urol Rep. 2021 Oct 7;22(10):49. doi: 10.1007/s11934-021-01065-7 (PMC8497313; doi:10.1007/s11934-021-01065-7)
Supplement: Supplementary file 1 — Supplementary file1 (DOCX 13 KB) [file 11934_2021_1065_MOESM1_ESM.docx]

COMMENTS FOR THE AUTHOR:

Dear Aleta,

Thank you for your comments

*1- Please restructure your Abstract into 3 sections: Reason for Review, Recent Findings, and Summary*

Absolutely, this has been restructured as suggested

*2- Please address the Corresponding Author issue -- either fix title page to Dr. Gravestock, or do Change of Authorship form to have Dr. Somani replace throughout the system*

Apologies for the confusion regarding this, I have attached a signed change of authorship form so that Professor Somani can be changed to the corresponding author.

*3- We need signed ICJME forms for each author. Section 6 on these forms will comprise a Conflict of Interest statement which you need to include in the paper along with a Human/Animal Studies informed consent statement:*

These have been completed and included.

*4- Section 6 on the ICJME forms, once collated,  will comprise a Conflict of Interest statement which you need to include in the paper (e.g. Drs. X and Y have nothing to disclose, Dr. Z declares this....). Please also include with a Human/Animal Studies informed consent statement of one of these two options:

    This article does not contain any studies with human or animal subjects performed by any of the authors.
    OR
    Human and Animal Rights and Informed Consent: All reported studies/experiments with human or animal subjects performed by the authors were performed in accordance with all applicable ethical standards including the Helsinki declaration and its amendments, institutional/national research committee standards, and international/national/institutional guidelines.*

Apologies for the oversight, this has now been included in the declarations section

*5 -- Can you please clarify if your figure(s)/table(s) is original or not? If not, you will need to obtain and submit the appropriate Permissions Notice and adjust the credit line in the caption as indicated within that Notice. *We cannot publish your paper without this.**

All of the included figures/table are original and I have added a line to the declarations section to state this.

*6 - Please annotate 3-5 recent references with * (Impt) or ** (very impt) and at the end of the reference include a few words as to its significance.*

Of course, the references have been reviewed and significant ones have been highlighted as suggested.

Apologies for the delays in returning this, We look forward to hearing from you in due course regarding our submission and would be more than happy to address any further questions or comments.

Kind Regards,

Paul Gravestock
