# Supplementary material for: Role of Ureteroscopy in Treatment of Upper Tract Urothelial Carcinoma
Source: Curr Urol Rep. 2021 Oct 7;22(10):49. doi: 10.1007/s11934-021-01065-7 (PMC8497313; doi:10.1007/s11934-021-01065-7)
Supplement: Supplementary file 2 — Supplementary file2 (PDF 1705 KB) [file 11934_2021_1065_MOESM2_ESM.pdf]

Change of authorship request form (pre-acceptance)

Please read the important information on page 4 before you begin

This form should be used by authors to request any change in authorship including changes in corresponding authors. Please fully complete all sections. Use black ink and block capitals and provide each author's full name with the given name first followed by the family name.

Please note: In author collaborations where there is formal agreement for representing the collaboration, it is sufficient for the representative or legal guarantor (usually the corresponding author) to complete and sign the Authorship Change Form on behalf of all authors.

Section 1: Please provide the current title of manuscript
(For journals: Please provide the manuscript ID, title and/or DOI if available.)
(For books: Please provide the title, ISBN and/or DOI if available.)

**Manuscript ID no. in case of unpublished manuscript:**	CUUR-D-21-00016
**DOI in case of published manuscript:**	
**ISBN (for books):**	

Title: ROLE OF URETEROSCOPY IN TREATMENT OF UPPER TRACT UROTHELIAL CARCINOMA

Section 2: Please provide the previous authorship, in the order shown on the manuscript before the changes were introduced. Please indicate the corresponding author by adding (CA) behind the name.

	First name(s)	Family name	ORCID or SCOPUS id, if available
1st author	Jeremy *	Ng Chieng Hin	
2nd author	Dinul *	Hettiarachchilage	0000-0003-1916-9298
3rd author	Paul	Gravestock (CA)	0000-0003-2343-3418
4th author	Bhavan	Rai	
5th author	Bhaskar	K Somani	0000-0002-6248-6478
6th author	Rajan	Veeratterapillay	
7th author			

Please use an additional sheet if there are more than 7 authors.

*Joint first authors

SPRINGER NATURE Springer Nature is one of the world's leading global research, educational and professional publishers, created in May 2015 through the combination of Nature Publishing Group, Palgrave Macmillan, Macmillan Education and Springer Science+Business Media.

Adis Bohn Stafleu van Loghum palgrave macmillan Springer

Change of authorship request form (pre-acceptance)

Section 3: Please provide a justification for change. Please use this section to explain your reasons for changing the authorship of your manuscript, e.g. what necessitated the change in authorship? Please refer to the (journal) policy pages for more information about authorship. Please explain why omitted authors were not originally included and/or why authors were removed on the submitted manuscript.

- Only change is corresponding author, incorrectly assigned on submission.

Section 4: Proposed new authorship. Please provide your new authorship list in the order you would like it to appear on the manuscript. Please indicate the corresponding author by adding (CA) behind the name. <u>If the corresponding author has changed, please indicate the reason under section 3.</u>

	First name(s)	Family name (this name will appear in full on the final publication and will be searchable in various abstract and indexing databases)
1st author	Jeremy *	Ng Chieng Hin
2nd author	Dinul *	Hettiarachchilage
3rd author	Paul	Gravestock
4th author	Bhavan	Rai
5th author	Bhaskar	K Somani (CA)
6th author	Rajan	Veeratterapillay
7th author		

Please use an additional sheet if there are more than 7 authors.

*Joint first authors

SPRINGER NATURE Springer Nature is one of the world's leading global research, educational and professional publishers, created in May 2015 through the combination of Nature Publishing Group, Palgrave Macmillan, Macmillan Education and Springer Science+Business Media.

Change of authorship request form (pre-acceptance)

Section 5: Author contribution, Acknowledgement and Disclosures. Please use this section to provide a new disclosure statement and, if appropriate, acknowledge any contributors who have been removed as authors and ensure you state what contribution any new authors made (if applicable per the journal or book (series) policy). Please ensure these are updated in your manuscript - after approval of the change(s) - as our production department will not transfer the information in this form to your manuscript.

**New acknowledgements:**<br><br><br>**New Disclosures (financial and non-financial interests, funding):**<br><br><br>**New Author Contributions statement (if applicable per the journal policy):**

State ‘Not applicable’ if there are no new authors.

SPRINGER NATURE Springer Nature is one of the world’s leading global research, educational and professional publishers, created in May 2015 through the combination of Nature Publishing Group, Palgrave Macmillan, Macmillan Education and Springer Science+Business Media.

Adis Bohn Stafleu van Loghum palgrave macmillan Springer

Change of authorship request form (pre-acceptance)

Section 6: Declaration of agreement. *All* authors, unchanged, new and removed *must* sign this declaration.
(NB: Please print the form, sign and return a scanned copy. Please note that signatures that have been inserted as an image file are acceptable as long as it is handwritten. Typed names in the signature box are unacceptable.) *** Please delete as appropriate. Delete all of the bold if you were on the original authorship list and are remaining as an author.**

	First name	Family name		Signature	Affiliated institute	Date
1st author	Jeremy	Ng Chieng Hin	I agree to the proposed new authorship shown in section 4 /~~**and the addition/removal*of my name to the authorship list**~~**.**	[illegible]	University of Newcastle Newcastle, UK	08/04/2021
2nd author	Dinul	Hettiarachchilage	I agree to the proposed new authorship shown in section 4 /~~**and the addition/removal*of my name to the authorship list**~~**.**	[illegible]	University of Newcastle Newcastle, UK	07/04/2021
3rd author	Paul	Gravestock	I agree to the proposed new authorship shown in section 4 /~~**and the addition/removal*of my name to the authorship list**~~**.**	[illegible]	Department of Urology, Freeman Hospital, Newcastle, UK	07/04/2021
4th authors	Bhavan	Rai	I agree to the proposed new authorship shown in section 4 /~~**and the addition/removal*of my name to the authorship list**~~**.**	[illegible]	Department of Urology, Freeman Hospital, Newcastle, UK	18/05/2021
5th author	Bhaskar	K Somani	I agree to the proposed new authorship shown in section 4 /~~**and the addition/removal*of my name to the authorship list**~~**.**	[illegible]	Department of Urology, University Hospital Southampton, UK	12/04/2021
6th author	Rajan	Veeratterapillay	I agree to the proposed new authorship shown in section 4 /~~**and the addition/removal*of my name to the authorship list**~~**.**	[illegible]	Department of Urology, Freeman Hospital, Newcastle, UK	19/04/2021
7th author			I agree to the proposed new authorship shown in section 4 /**and the addition/removal*of my name to the authorship list.**			

Please use an additional sheet if there are more than 7 authors.

SPRINGER NATURE Springer Nature is one of the world's leading global research, educational and professional publishers, created in May 2015 through the combination of Nature Publishing Group, Palgrave Macmillan, Macmillan Education and Springer Science+Business Media.

Change of authorship request form (pre-acceptance)

Important information. Please read.

- Please return this form, fully completed, to Springer Nature. We will consider the information you have provided to decide whether to approve the proposed change in authorship. We may choose to contact your institution for more information or undertake a further investigation, if appropriate, before making a final decision.

- By signing this declaration, all authors guarantee that the order of the authors are in accordance with their scientific contribution, if applicable as different conventions apply per discipline, and that only authors have been added who made a meaningful contribution to the work.

- Please note, we cannot investigate or mediate any authorship disputes. If you are unable to obtained agreement from all authors (including those who you wish to be removed) you must refer the matter to your institution(s) for investigation. Please inform us if you need to do this.

- If you are not able to return a fully completed form within **30 days** of the date that it was sent to the author requesting the change, we may have to withdraw your manuscript. We cannot publish manuscripts where authorship has not been agreed by all authors (including those who have been removed).

- Incomplete forms will be rejected.

SPRINGER NATURE Springer Nature is one of the world's leading global research, educational and professional publishers, created in May 2015 through the combination of Nature Publishing Group, Palgrave Macmillan, Macmillan Education and Springer Science+Business Media.
