# Supplementary figures and images for: Role of Ureteroscopy in Treatment of Upper Tract Urothelial Carcinoma
Source: Curr Urol Rep. 2021 Oct 7;22(10):49. doi: 10.1007/s11934-021-01065-7 (PMC8497313; doi:10.1007/s11934-021-01065-7)

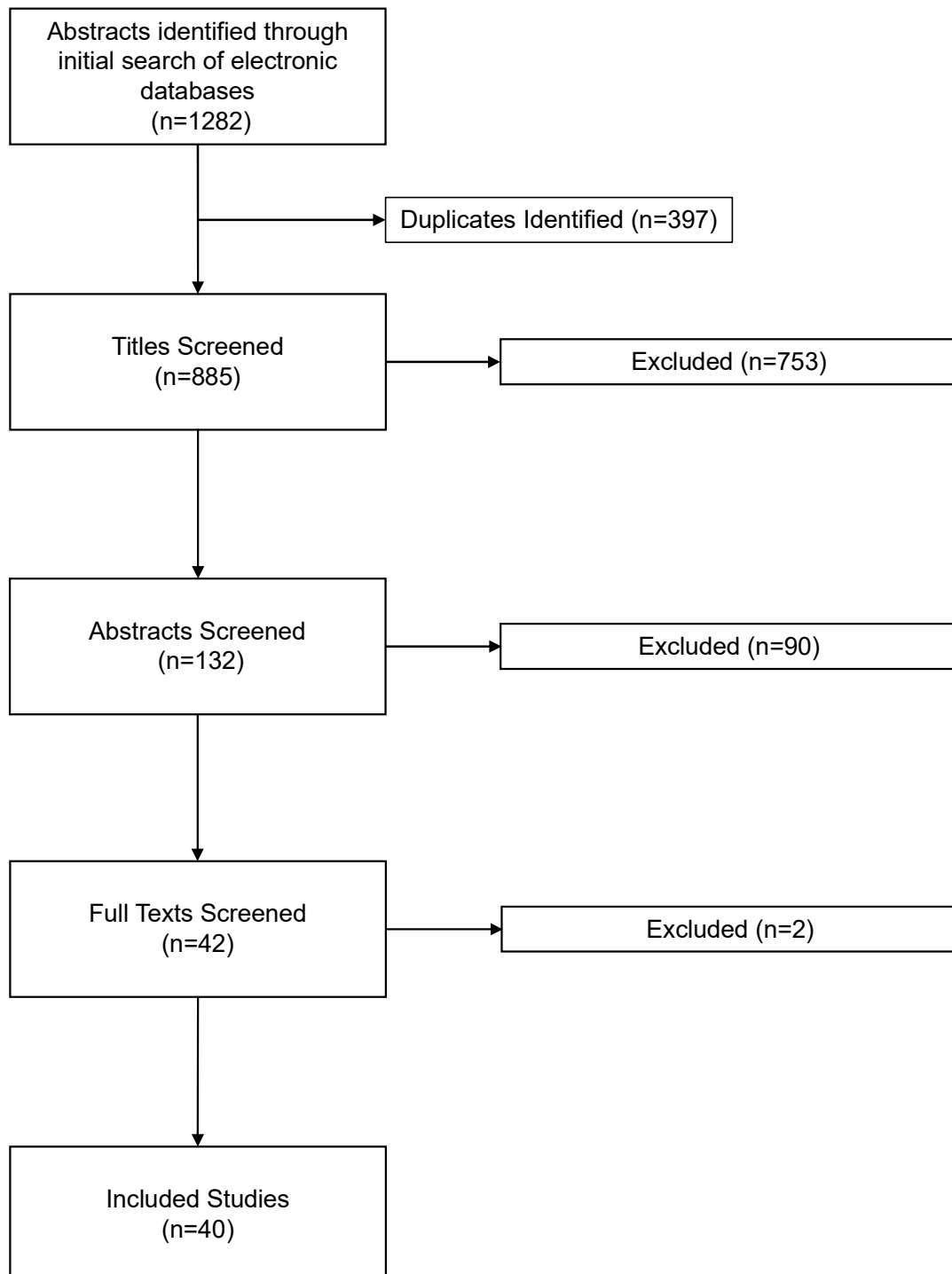

Supplement: Supplementary file 9 — Supplementary file9 (PDF 93 KB) [file 11934_2021_1065_MOESM9_ESM.pdf]
